# Nuclear poly(A) binding protein 1 (PABPN1) mediates zygotic genome activation-dependent maternal mRNA clearance during mouse early embryonic development

**DOI:** 10.1093/nar/gkab1213

**Published:** 2021-12-14

**Authors:** Long-Wen Zhao, Ye-Zhang Zhu, Yun-Wen Wu, Shuai-Bo Pi, Li Shen, Heng-Yu Fan

**Affiliations:** MOE Key Laboratory for Biosystems Homeostasis, Life Sciences Institute, Zhejiang University, Hangzhou 310058, China; MOE Key Laboratory for Biosystems Homeostasis, Life Sciences Institute, Zhejiang University, Hangzhou 310058, China; MOE Key Laboratory for Biosystems Homeostasis, Life Sciences Institute, Zhejiang University, Hangzhou 310058, China; MOE Key Laboratory for Biosystems Homeostasis, Life Sciences Institute, Zhejiang University, Hangzhou 310058, China; MOE Key Laboratory for Biosystems Homeostasis, Life Sciences Institute, Zhejiang University, Hangzhou 310058, China; MOE Key Laboratory for Biosystems Homeostasis, Life Sciences Institute, Zhejiang University, Hangzhou 310058, China

## Abstract

An embryo starts its life with maternal mRNA clearance, which is crucial for embryonic development. The elimination of maternal transcripts occurs by the joint action of two pathways: the maternally encoded mRNA decay pathway (M-decay) and the zygotic genome activation (ZGA)-dependent pathway (Z-decay). However, zygotic factors triggering maternal mRNA decay in early mammalian embryos remain largely unknown. In this study, we identified the zygotically encoded nuclear poly(A) binding protein 1 (PABPN1) as a factor required for maternal mRNA turnover, with a previously undescribed cytoplasmic function. Cytoplasmic PABPN1 docks on 3′-uridylated transcripts, downstream of terminal uridylyl transferases TUT4 and TUT7, and recruits 3′-5′ exoribonuclease DIS3L2 to its targets, facilitating maternal mRNA decay. *Pabpn1*-knockout in mice resulted in preimplantation stage mortality due to early developmental arrest at the morula stage. Maternal mRNAs to be eliminated via the Z-decay pathway failed to be removed from *Pabpn1*-depleted embryos. Furthermore, PABPN1-mediated Z-decay is essential for major ZGA and regulates the expression of cell fate-determining factors in mouse preimplantation embryos. This study revealed an unforeseen cytoplasmic function of PABPN1 coupled with early embryonic development, characterized the presence of a zygotic destabilizer of maternal mRNA, and elucidated the Z-decay process mechanisms, which potentially contribute to human fertility.

## INTRODUCTION

The maternal-to-zygotic transition (MZT), fundamental to all animals, occurs via a combination of two phases, the clearance of maternal RNAs and proteins, followed by the gradual zygotic genome activation (ZGA), during which protein synthesis switches from maternal products to proteins synthesized by the embryonic nucleus (1–3). The primary event that initiates this transition is eliminating an abundant cohort of mRNAs conferred by the mother in a highly coordinated manner (1). The degradation has been well characterized in *Drosophila*, zebrafish and *Xenopus*, and is achieved by two continuous activities: the former is exclusively mediated by maternally provided gene products and is known as M-decay; the latter is performed by newly expressed zygotic products, therefore called Z-decay (4–6). Maternal mRNA clearance is crucial to determine the developmental competence of fertilized eggs, and failure in this clearance causes developmental arrest across different species (7–11).

Poly(A) tails are non-templated additions of adenosines at the 3′-end of most eukaryotic mRNAs, which play pivotal roles in mRNA stability (12). Deadenylation is the rate-limiting step during mRNA degradation (13). Recent research uncovered that critical factors required for maternal mRNA turnover are vastly interconnected with deadenylation in mammals. CNOT6L, a catalytic subunit of the CCR4-NOT deadenylase complex, is associated with ZFP36L2 in mediating maternal mRNA decay during oocyte maturation (14). An oocyte-specific poly(A)-binding protein nuclear 1 like factor (PABPN1L) has been identified, which functions as a poly(A)-binding adapter for the mammalian MZT licensing factor BTG4, coupled with CCR4-NOT deadenylase, to facilitate maternal mRNA clearance during the MZT (9,15). All of these factors are expressed in oocytes and are responsible for stage-specific degradation of maternal mRNAs during M-decay.

The Z-decay pathway has also been characterized in murine and human maternal mRNA degradation profiling (10,11). The maternally deposited BTG4 and the CCR4-NOT deadenylase complex continue to serve for Z-decay, and the zygotically expressed TUT4 and TUT7 terminal uridylyl transferases catalyze terminal uridylation of maternal transcripts after deadenylation, facilitating maternal mRNA clearance (10,16). However, these zygotic factors account for a limited part of the Z-decay process; additional mRNA destabilizers and pathways that coordinate the steps that follow 3′-uridylation remain poorly understood.

Poly(A) and oligo(U) are regarded as antagonistic players in controlling mRNA stability. The poly(A) tails are coated with poly(A)-binding proteins all along, which stabilize poly(A) RNA and facilitate translation (17,18). In turn, oligo(U) tails appear as general markers of global mRNA decay, occurring on PABP-free mRNAs (19). Nevertheless, the traditional roles comprise a key section of the mRNA life, and recent research has improved our understanding of the interplay between poly(A) and oligo(U). TUT4 and TUT7 have been demonstrated to target not only mRNAs lacking poly(A) tails but also mRNAs with short poly(A) tails (approximately < 25 nt) (16,19–22). A contradictory role for cytoplasmic PABP in activating deadenylation was revealed (23,24). In addition, the cytoplasmic mRNA degradation is also elucidated in the newly identified nuclear PABP during the MZT (15). These findings imply the presence of intricate networks of mRNA turnover, which remain to be fully elucidated.

In this study, we identify PABPN1 as a zygotic factor mediating Z-decay in a novel cytoplasmic pathway during mouse MZT. *Pabpn1*-knockout murine embryos are lethal at the morula stage due to impaired maternal mRNA clearance after fertilization. PABPN1 recruits DIS3 like 3′-5′ exoribonuclease 2 (DIS3L2), a specific cytoplasmic exoribonuclease of uridylated mRNAs, to TUT4/7-mediated 3′-oligouridylated transcripts to facilitate their decay. This study unveils a previously unidentified role of PABPN1 in the progression of early embryo development and contributes to elucidating the process of maternal mRNA decay in mammals.

## MATERIALS AND METHODS

### Animals

C57B6 background mouse strains were used in this study. The CRISPR-CAS9 knockout *Pabpn1^fl/fl^* mice generation strategy is illustrated in Supplementary Figure S1A. *Pabpn1^fl/fl^*; *Gdf9-Cre* mice were produced by crossing mice carrying the *Pabpn1^fl^* allele with previously reported *Gdf9-Cre* transgenic mice (9), as illustrated in Supplementary Figure S1B. Mice genomic tail DNA was extracted to perform PCR genotyping. All primers used in this study are listed in Supplementary Table S1. Mice were bred under specific germ-free conditions in a controlled environment of 20–22ºC, with a 12/12 h light/dark cycle, 50–70% humidity, and food and water provided *ad libitum*. Animals were carefully treated according to the Animal Research Committee guidelines of Zhejiang University.

### Superovulation and fertilization

Female mice (21–23 days old) were intraperitoneally injected with 5 IU of pregnant mare's serum gonadotropin (PMSG). After 44 h, mice were then injected with 5 IU of human chorionic gonadotropin (hCG) and mated with adult males. The presence of vaginal plugs confirmed successful mating. Embryos were harvested from oviducts at the indicated times post-hCG injection.

### Oocyte and embryo collection and *in vitro* culture

Female mice at 21–23 days of age were injected with 5 IU of PMSG and humanely euthanized 44 h later for harvesting oocytes. Zygotes were harvested as described above. Oocytes at the germ-vesicle (GV) stage and zygotes were harvested in M2 medium (Sigma-Aldrich; M7167). Oocytes were cultured in M16 medium (Sigma-Aldrich; M7292), and zygotes were cultured in KSOM medium (Sigma-Aldrich; MR-101-D). The medium was covered with mineral oil at 37°C in a 5% CO_2_ atmosphere.

### Microinjection

All microinjections were performed using an Eppendorf transferman NK2 micromanipulator. Fully grown GV oocytes were harvested in M2 medium with 2 μM milrinone to inhibit spontaneous GV breakdown. Zygotes were obtained by superovulation of 7- to 8-week-old females mated with males of the same strain and harvested in M2 medium. Denuded oocytes or zygotes with well-recognized pronuclei were injected with 5 to 10 pl siRNA (20 μM) into the cytoplasm. After injection, the oocytes were washed and cultured in M2 medium plus 2 μM milrinone at 37°C with 5% CO_2_; the zygotes were washed and cultured in KSOM medium at 37°C with 5% CO_2_ and covered with mineral oil at 37°C in a 5% CO_2_ atmosphere. The siRNA sequences used are listed in Supplementary Table S1.

### Western blot (WB) analysis

Oocytes/embryos were collected and lysed in SDS loading buffer and then denatured for 5 min at 95°C. Total extracted oocyte proteins were separated by the SDS-PAGE method and transferred to PVDF membranes using a semi-dry transfer apparatus. The PVDF membranes were blocked in TBST buffer containing 5% skimmed milk for 30 min. The target protein was probed with primary antibodies and an HRP-linked secondary antibody. The bound antibodies were detected using the Super Signal West Femto maximum sensitivity substrate. The primary antibodies and dilution factors used are listed in Supplementary Table S2.

### Cell culture, plasmid transfection and immunoprecipitation

HeLa cells were obtained from the American Type Culture Collection and were recently authenticated and tested for mycoplasma contamination. Cells were cultured in DMEM plus 10% fetal bovine serum and 1% penicillin-streptomycin solution at 37°C in a humidified incubator supplemented with 5% CO_2_. Human *Pabpn1*, *Dis3l2* clones were picked out from the human ORF library and cloned into an N-terminal FLAG- or HA- vector. The indicated mutants were generated by PCR-based direct mutagenesis and confirmed by Sanger sequencing. For transient expression, plasmids were transfected using Lipofectamine 2000 (Invitrogen). Cells were then harvested in a lysis buffer (50 mM Tris–HCl, pH 7.5, 150 mM NaCl, 10% glycerol and 0.5% NP-40) after 48 h of transfection. After centrifugation, the supernatant was subjected to immunoprecipitation with different affinity agarose gels (Sigma). The bead-bound proteins were eluted using an SDS sample buffer for WB analysis.

### Preparation of cell extracts

Whole-cell extracts, nuclear extracts, and cytoplasmic extracts were prepared as previously described (25). Briefly, to prepare nuclear extracts, treated cells were placed on ice and washed once with cold PBS. Cells were then scraped off the dish and collected by centrifugation at 1500 × g for 5 min. The cell pellet was resuspended in 5 ml cell lysis buffer (10 mM HEPES, pH 7.9, 1.5 mM MgCl_2_, 10 mM KCl, 0.5 mM dithiothreitol and 0.2 mM phenylmethylsulfonyl fluoride) and centrifuged at 1500 × *g* for 5 min. Cells were resuspended in 2 times the original packed cell volume of cell lysis buffer, allowed to swell on ice for 10 min, and homogenized with 10 strokes of a Dounce homogenizer (B pestle). Nuclei fractions were collected by centrifugation at 3300 × g for 15 min at 4°C, and the supernatant was saved for cytoplasmic extracts. The nuclei were resuspended, using six strokes of a Teflon-glass homogenizer, in 3 volumes of nuclear extraction buffer (20 mM HEPES, pH 7.9, 1.5 mM MgCl_2_, 400 mM KCl, 0.5 mM dithiothreitol, 0.2 mM phenylmethylsulfonyl fluoride and 25% glycerol). The nuclear suspension was stirred on ice for 30 min and then centrifuged at 89 000 × g for 30 min. The supernatant was collected and concentrated in a Microcon 10 concentrator by centrifugation at 14 000 × g for 3 h at 4°C. For the preparation of cytoplasmic extracts, the supernatant obtained after removal of nuclei was mixed thoroughly with 0.11 volume of 10 × cytoplasmic extraction buffer (1 × cytoplasmic extraction buffer: 30 mM HEPES, pH 7.9, 140 mM KCl, 3 mM MgCl_2_) and then centrifuged at 89 000 × g for 1 h. The supernatant was collected and concentrated in a Microcon-10 concentrator via centrifugation at 14 000 × g for 1 h at 4°C. The concentrated protein extract was mixed with SDS sample buffer for WB analysis.

### RNA-seq analysis

To generate RNA-seq data, embryonic mRNA transcripts were amplified using the Smart-seq2 protocol (26). Briefly, each sample including 10 embryos was lysed in 2 μl lysis buffer (0.2% Triton X-100 and 2 IU/μl RNase inhibitor) and followed by reverse-transcription using the SuperScript III reverse transcriptase and 10-cycle PCR amplification. RNA samples were sequenced on the Illumina HiSeq platform as paired-end 150-base reads. Raw reads were trimmed with Trimmomatic-0.36 to 50 bp and mapped to the mouse genome (mm9) with the TopHat (v2.0.11) software. The mapped reads were subsequently assembled into transcripts guided by reference annotation (University of California at Santa Cruz [UCSC] gene models) with Cufflinks version 2.2.1. The expression level of each gene was quantified and indicated as normalized FPKM based on the FPKM of exogenous External RNA Controls Consortium (ERCC) (Invitrogen, Cat. No. 4456740) transcript mixtures. Genes with FPKM < 1 in all sampls were excluded, and for the remaining genes, all FPKM values smaller than 1 were set to 1 in subsequent analyses.

Statistical analyses were performed using the R platform (http://www.rproject.org). The Spearman correlation coefficient (rs) was calculated using the ‘cor’ function, and the complete linkage hierarchical algorithm was used to cluster the genes. Quality controls of RNA-seq results are provided in Supplementary Table S3. All the FPKMs of the RNA-seq results are listed in Supplementary Table S4.

### RNA isolation and RT-qPCR analysis

Total RNA extraction was performed using the RNeasy Mini Kit (Qiagen, Cat. No. 74106), and cDNA was subsequently created using SuperScript III reverse transcriptase (Invitrogen; Cat. No. 18080200). Quantitative PCR (qPCR) was run on an Applied Biosystems 7500 Real-Time PCR System using a Power SYBR Green PCR Master Mix. Relative mRNA levels were calculated by normalizing the levels of endogenous glyceraldehyde 3-phosphate dehydrogenase (*Gapdh*) mRNA used as a control. The relative expression level and fold change were calculated using following equations:

$$\begin{equation*}{\rm{Relative\, expression\, level (}}\Delta {{\rm{C}}_{\rm{T}}}{\rm{)}} = {{\rm{C}}_{{\rm{T(gene)}}}} - {{\rm{C}}_{{\rm{T(}}Gapdh{\rm{)}}}}{\rm{;}}\end{equation*}$$

$$\begin{equation*}\Delta \Delta {{\rm{C}}_{\rm{T}}} = \Delta {{\rm{C}}_{{\rm{T(knockdown)}}}} - \Delta {{\rm{C}}_{{\rm{T(WT)}}}};\end{equation*}$$

$$\begin{equation*} {\rm{Fold\, change}} = 2^{-\Delta \Delta {\rm CT}}. \end{equation*}$$

For each experiment, qPCR reactions were carried out in independent biological triplicate. Primer sequences are listed in Supplementary Table S1.

### 3′-Ligation RACE

Each sample included 100 2-cell embryos. Total RNA was isolated using RNeasy Mini Kit (Qiagen, Cat. No. 74106) and ligated overnight at 25°C with a 2 μM Universal miRNA Cloning Linker (NEB, Cat. No. S1315S) using 200 U T4 RNA ligase 2 truncated KQ (NEB, Cat. No. M0373S) in the presence of 25% PEG 8000 and RNaseOUT. The ligated RNAs were purified using RNA Clean & Concentrator-25 columns (Zymo Research, Cat. No. R1017), and reverse transcribed using SuperScript III (Invitrogen; Cat. No. 18080200) and universal RT+ linker primer (Supplementary Table S1). *Gm4745* mRNA tails were amplified using *Gm4745*-specific forward and universal RT+ linker reverse primers (Supplementary Table S1). PCR products were visualized on 2% agarose gels (Supplementary Figure S3D), extracted, cloned into pGEM-T easy vectors (Promega), and sequenced by Sanger DNA sequencing.

### Ribonucleoprotein immunoprecipitation (RIP) assay

The RIP assay procedure was adapted from a previously reported method with modification (27). Briefly, HeLa cells were collected and lysed in RIP lysis buffer (50 mM Tris–HCl pH 7.4, 1% Triton X-100, 150 mM NaCl, 5 mM EDTA, protease inhibitor cocktail and RNase inhibitor). A total of 10% of the cell lysate supernatant was used as the ‘input’, while 90% was subjected to immunoprecipitation with FLAG agarose gels; after incubation at 4°C for 4 h, bead-bound RNAs were eluted and extracted using the RNeasy Mini Kit (Qiagen, 74106) and used for reverse-transcription using M-MLV Reverse Transcriptase (Invitrogen). Relative cDNA abundance was analyzed by qPCR.

### Immunofluorescence

Embryos were fixed in 4% paraformaldehyde in PBS for 30 min and permeabilized in PBS containing 0.2% Triton X-100 for 20 min. After being blocked with 1% bovine serum albumin in PBS, the oocytes were incubated with appropriate primary antibodies for 1 h and sequentially labeled with Alexa Fluor 594- or 488-conjugated secondary antibodies and 4′,6-diamidino-2-phenylindole (DAPI) for 30 min. Fluorescent images of embryos were taken on a Zeiss LSM710 confocal microscope. Equal exposure time was used to capture images in the same experiment. Signal quantification was conducted using ImageJ 1.33u software (National Institutes of Health). Fluorescence of the antibodies (S_a_), background fluorescence of the antibodies (S_ba_), fluorescence of DNA (S_d_), background fluorescence of DNA (S_bd_), area of the Sa (A_a_) and area of the Sd (A_d_) were measured. The antibody signal was quantified as:

$$\begin{equation*}{\rm{S}} = \left[ {{{\rm{A}}_{\rm{d}}}\left( {{{\rm{S}}_{\rm{a}}} - {{\rm{S}}_{{\rm{ba}}}}} \right)/{{\rm{A}}_{\rm{a}}}\left( {{{\rm{S}}_{\rm{d}}} - {{\rm{S}}_{{\rm{bd}}}}} \right)} \right].\end{equation*}$$

The antibodies used are listed in Supplementary Table S2.

### Detection of protein synthesis in embryos

To measure protein synthesis in embryos, all the embryos were cultured in KSOM medium supplemented with 50 μM l-homopropargylglycine (HPG) for 1 h, and HPG was detected using a Click-iT^®^ HPG Alexa Fluor^®^ Protein Synthesis Assay Kit (Life Technologies) according to the user manual. The mean cytoplasmic signal was quantified based on the signal from the middle of each embryo and quantified in ImageJ.

### Statistical analysis

Results in this study were reported as means and standard errors (SEM). Each experiment included at least three independent samples and was repeated at least three times. A two-tailed unpaired Student's *t*-tests were used to analyze the results for comparing two experimental groups. Statistically significant values of *P* < 0.05, *P* < 0.01 and *P* < 0.001 were indicated by asterisks (*), (**) and (***), respectively. All tests and p values were provided in the corresponding legends and/or figures.

## RESULTS

### PABPN1 is an early expressed zygotic factor in early mammalian embryos

**Figure 1.**
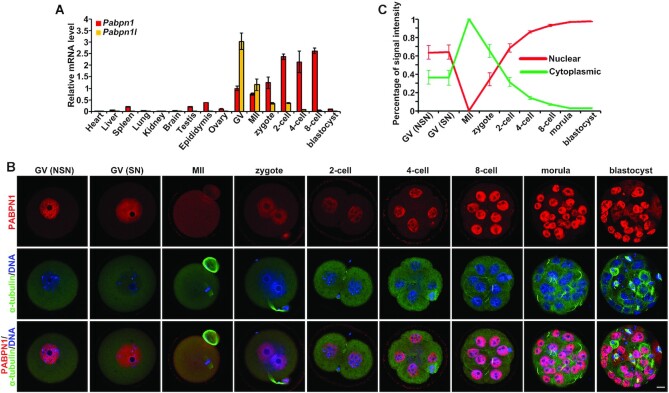
PABPN1 expression in mouse preimplantation embryos. (**A**) RT-qPCR results showing mRNA levels of *Pabpn1* and *Pabpn1l* in mouse tissues and oocytes/embryos. The *Pabpn1* mRNA level in germinal vesicle (GV) stage oocytes was set to 1.0. Error bars, SEM. *n* = 3 biological replicates. (**B**) Confocal microscopic images of PABPN1 (red) and α-tubulin (green) immunofluorescence in mouse oocytes and preimplantation embryos. DNA was counterstained with DAPI (blue). At each stage, more than 10 oocytes or embryos were examined, with similar results. Scale bar = 10 μm. (**C**) The proportion of PABPN1 signals in cytoplasm and nuclei in oocytes or blastomeres. Error bars, S.E.M. *n* = 10 oocytes or embryos at each stage.

The MZT appears as a primary switch in the transcriptome. To identify the key factors involved in the transition, we looked into the mRNA-binding proteins (mRBPs) profiling during the zebrafish MZT using mRNA interactome capture (28) and noticed that PABPN1 was detected specifically at the onset of zygotic genome activation (ZGA). Quantitative RT-PCR (RT-qPCR) from mouse oocytes and early embryos showed that *Pabpn1* was highly expressed during ZGA in comparison to its homolog *Pabpn1l* (Figure 1A) (15), suggesting that the presence of a conserved role of PABPN1 in early embryos across species. In contrast to other cell types, *Pabpn1* and *Pabpn1l* are preferentially expressed in oocytes and preimplantation embryos, however, with a distinct pattern. The level of *Pabpn1l* mRNA was higher than that of *Pabpn1* mRNA in oocytes and quickly decreased to an undetectable level after fertilization. However, *Pabpn1* mRNA levels were significantly increased after ZGA, remained relatively high until the 8-cell stage, and then dropped at the blastocyst stage (Figure 1A), which is in line with the results of previous studies and indicates that PABPN1L is a maternally deposited factor acting as a placeholder of mRNA polyA tails during the MZT. At the same time, PABPN1 is a zygotically synthesized factor that may play a role in early embryos.

Immunofluorescent staining confirmed the gradual accumulation of PABPN1 during early embryo development; however, the subcellular distribution varied depending on the stage of development. After germinal vesicle breakdown (GVBD), PABPN1 was spread throughout the cytoplasm in metaphase II (MII) oocytes. Following fertilization, the male and female pronuclei were formed, and the cytosolic PABPN1 initiated a trend of translocation from the cytoplasm to the nucleus. Even so, the cleaved embryos still deposited a part of PABPN1 in the cytoplasm until the 8-cell stage (Figure 1B, C).

### PABPN1 is crucial for early embryo development

**Figure 2.**
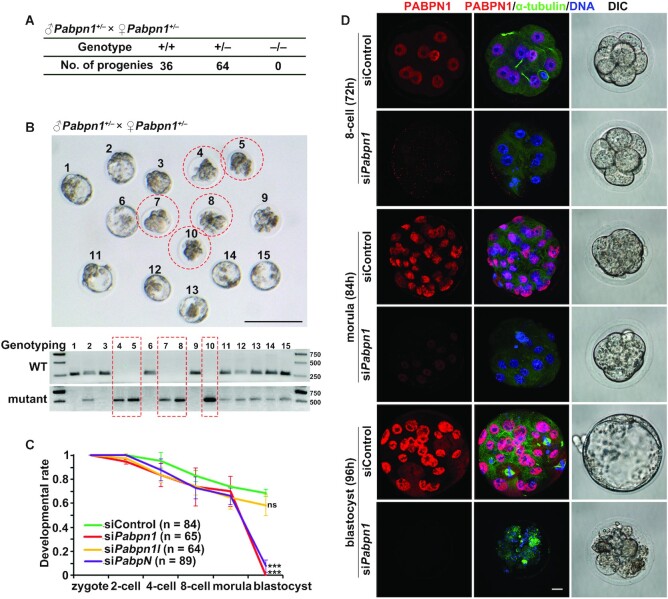
PABPN1 is crucial for mouse early embryo development. (**A**) Genotyping results for *Pabpn1*^+/+^, *Pabpn1*^+/−^ and *Pabpn1*^−/−^ mouse offspring. No *Pabpn1*^−/−^ pups were obtained after birth. (**B**) Representative images (upper panel) and genotyping results (lower panel) showing the embryos at day 4.5 post-coitus (dpc) collected from *Pabpn1^+/^^−^* female mice, which were mated with adult *Pabpn1^+/^^−^* male mice. Note that *Pabpn1^/^^−^^/^^−^* embryos were circled by a dashed line. Scale bars = 100 μm. (**C**) Zygotes collected from oviducts were microinjected with the indicated siRNAs and then cultured for 96 h. The developmental rates of embryos at the indicated stages were evaluated. The number of analyzed embryos was indicated (n). Error bars, S.E.M. ***P< 0.001 by two-tailed Student's *t*-test. ns: non-significant. (**D**) PABPN1 (red) and α-tubulin (green) immunofluorescence in siControl- or si*Pabpn1-*microinjected embryos at the indicated time points after superovulation treatment. The embryos collected for imaging by differential interference contrast (DIC) microscope were at the same stages as the embryos showed in immunofluorescence. *n* = 8 embryos at each time point. Scale bar = 10 μm.

To study the *in vivo* function of *Pabpn1*, a CRISPR/Cas9-based strategy was utilized to create *Pabpn1*-knockout mice. We first generated a *Pabpn1*-floxed mouse strain (*Pabpn1^fl^^/+^)*, in which exons 3 and 4 of *Pabpn1* were flanked by 34 bp flox sequences (Supplementary Figure S1A). Then, we deleted *Pabpn1* in oocytes by crossing *Pabpn1^fl^^/+^* mice with *Gdf9-Cre* transgenic mice (Supplementary Figure S1A, B). After that, *Pabpn1^fl^^/+^; Gdf9-Cre* females were mated with wild-type (WT) males to obtain *Pabpn1^+/^^−^* mice. The deletion of exons 3 and 4 introduced a reading-frame shift and created a premature stop codon in the *Pabpn1* mRNA (Supplementary Figure S1A). Finally, we crossed *Pabpn1^+/^^−^* male and female mice to complete *Pabpn1* deletion (Supplementary Figure S1B). However, no *Pabpn1^−^^/^^−^* pups were obtained from this crossing, as detected from DNA genotyping, and the ratio of *Pabpn1^+/+^*, *Pabpn1^+/^^−^* pups was close to 1:2 (Figure 2A). To obtain *Pabpn1^−^^/^^−^* embryos, we analyzed genotypes of the embryos at 4.5 days post-coitus (dpc) (Figure 2B). Most of the embryos developed into blastocysts; however, the embryos circled out by the red dashed line is shown in Figure 2B (upper panel) were arrested at the morula stage. DNA genotyping results showed that those embryos that did not develop into blastocysts and were arrested at the morula stage were *Pabpn1^−^^/^^−^* embryos (Figure 2B; lower panel). Based on these results, we concluded that knockout of *Pabpn1* leads to early embryonic death.

In view of the limited amount of *Pabpn1^−^^/^^−^* embryos obtained from crossing and those further analyses could not be performed due to the processing done for DNA genotyping, we determined PABPN1 function in preimplantation development employing RNA interference (RNAi). This is initiated by injecting small double-stranded RNAs, small-interfering RNAs (siRNAs), which target mRNAs for degradation in a sequence-specific (29). After injecting siRNAs that target the *Pabpn1* gene into the cytoplasm of WT zygotes, the deficiency in *Pabpn1* transcripts in si*Pabpn1*-injected embryos was confirmed by RT-qPCR and immunofluorescence assays, suggesting the high efficiency of the si*Pabpn1*-mediated gene silencing (Supplementary Figure S1C and Figure 2D). Consistent with what was observed for the *Pabpn1^−^^/^^−^* embryos, the development of *Pabpn1*-knockdown embryos was halted at the morula stage (Figure 2C, D and Supplementary Figure S1D). To further confirm the roles of PABPN1 in early embryogenesis, we tested the possibility that PABPN1L is functionally redundant to PABPN1 by performing RNAi depletion of *Pabpn1l* or *PabpN* (both *Pabpn1* and *Pabpn1l*) from the zygote. *Pabpn1l* knockdown embryos successfully developed into blastocysts, but *Pabpn1*/*Pabpn1l* double knockdown embryos exhibited a morula-arrested phenotype similar to what was observed with *Pabpn1* deletion alone (Figure 2C and Supplementary Figure S1C, D). Together, these results indicated that PABPN1, rather than PABPN1L, is required for early embryonic development.

### *Pabpn1* depletion causes irregular Z-decay mRNAs accumulation and ZGA defects

**Figure 3.**
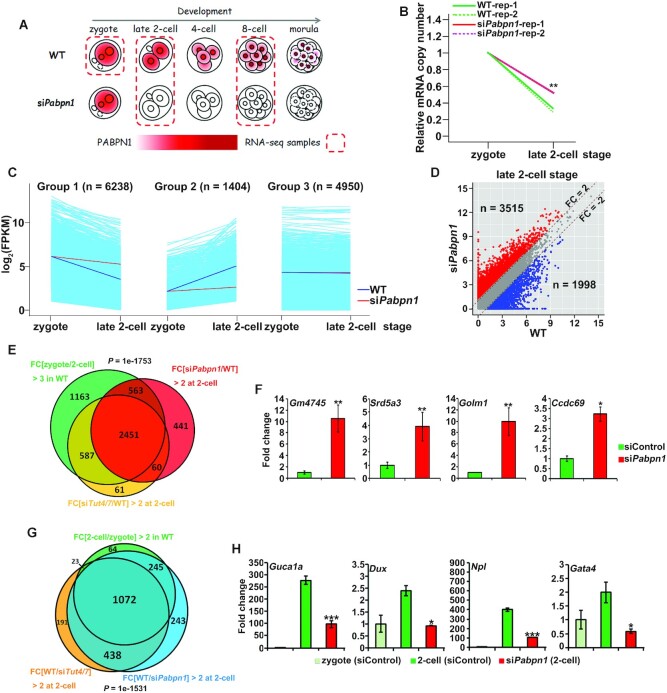
Transcriptome analyses in *Pabpn1*-depleted embryos during the MZT. (**A**) Schematic diagram showing the samples used for RNA-seq, circled by a dashed line. Zygotes, late 2-cell, and 8-cell embryos were collected from *in vivo* at 28, 43 and 72 h post-hCG injection. The red represents the subcellular localization and expression level of PABPN1. (**B**) Changes in relative mRNA copy numbers in control and *Pabpn1*-depleted embryos during the zygote-to-2-cell transition. Total mRNA copy numbers were calculated by normalizing with the ERCC spike-in, and mRNA expression at the zygote stage was set to 1.0. **P< 0.01 by two-tailed Student's *t*-test. (**C**) Classification of transcripts according to the expression pattern changes during the zygote-to-2-cell transition in WT embryos into three groups. Group 1: FPKM (zygote/2-cell) > 2; Group 2: FPKM (2-cell/zygote) > 2; Group 3: FPKM (zygote/2-cell) < 2 and FPKM (2-cell/zygote) <2. Each light blue line represents the expression level of one gene in WT embryos. The middle blue line and red line indicate the median expression level of the group in WT and si*Pabpn1* embryos, respectively. (**D**) Scatter plot showing changes in transcript levels in 2-cell embryos after *Pabpn1* depletion. Transcripts whose levels increased or decreased by more than 2-fold in *Pabpn1*-depleted embryos are highlighted in red or blue, respectively. (**E**) Venn diagrams showing the overlap of the upregulated transcripts in *Pabpn1*-depleted embryos (FPKM (si*Pabpn1*/WT) > 2 in 2-cell embryos) and the degraded transcripts from the zygote to 2-cell embryos in WT (FPKM (zygote/2-cell) > 3 in WT), as well as the upregulated transcripts in *Tut4/7*-depleted embryos (FPKM (si*Tut4/7*/WT) > 2 in 2-cell embryos). *P*= 1e-1753 by two-tailed Student's t-test. (**F**) RT-qPCR results showing the relative mRNA levels of the indicated Z-decay transcripts in 2-cell embryos with or without *Pabpn1* depletion. Error bars, S.E.M. *P< 0.05, **P< 0.01 by two-tailed Student's *t*-test. *n* = 3 biological replicates. (**G**) Venn diagram showing the overlap in transcripts whose levels increased during the zygote-to-2-cell transition in WT (FPKM (2-cell/zygote) > 2) and those whose levels decreased in *Pabpn1*-depleted embryos compared with those in WT at the 2-cell stage (FPKM (WT/si*Pabpn1* > 2 in 2-cell embryos), as well as transcripts whose levels decreased in *Tut4/7*-depleted embryos (FPKM (WT/si*Tut4/7*) > 2 in 2-cell embryos). *P*= 1e–1531 by two-tailed Student's *t*-test. (**H**) RT-qPCR results showing relative levels for the indicated zygotic genome activation (ZGA) transcripts in 2-cell embryos with or without *Pabpn1* depletion. Error bars, S.E.M. *P< 0.05, ***P< 0.001 by two-tailed Student's *t*-test. *n* = 3 biological replicates.

To determine the role of PABPN1 during early embryogenesis, we applied RNA-seq combined with RNAi at the zygote stage, which depleted *Pabpn1* after fertilization, and we subjected late 2-cell and 8-cell stage embryos to mRNA profiling (Figure 3A). Gene expression levels were determined by fragments per kilobase of transcripts per million mapped reads (FPKM), and the relative mRNA copy number was evaluated using the ERCC spike-in. All samples were analyzed in duplicate and exhibited a high correlation (Supplementary Table S5). Upon *Pabpn1* depletion, the global transcripts accumulated to abnormally high levels at the late 2-cell stage (Figure 3B). Two major developmental events occur during the MZT: the redundant maternal transcripts elimination and the transcription of the zygotic genome establishment (2). Based on these dynamic changes, we classified 12 592 genes into three groups (Figure 3C). Group 1 comprised 6238 transcripts decreasing throughout the zygote and late 2-cell stage (Figure 3C; left panel). Thus, the transcripts in this group were considered the maternal mRNAs that should be removed during the MZT. In comparison, group 2 contained 1,404 transcripts, showed ascended steadily in mRNA levels during the zygote-to-2-cell transition (Figure 3C; middle panel), which were deemed activated zygotic transcripts. Group 3 displayed no fluctuation across two stages, and comprised 4,950 mRNAs (Figure 3C; right panel). In *Pabpn1*-depleted embryos, the median levels of group 1 mRNAs were increased by 2.32-fold compared to those of the WT, whereas those of group 2 mRNAs decreased by 4.37-fold (Figure 3C). Thus, *Pabpn1* deficiency blocked both the degradation of maternal mRNAs and the activation of the zygotic genome during the MZT.

Further transcriptome analysis revealed that 3515 and 1998 transcripts were upregulated and downregulated by more than 2-fold in *Pabpn1*-depleted 2-cell embryos, respectively (Figure 3D). Analysis of the accumulated transcripts by gene set enrichment revealed that 3014 out of 3515 (85.7%) transcripts belonged to the previously identified Z-decay transcripts (10). Previously, we have reported that TUT4/7-mediated 3′-oligouridylation of maternal mRNAs is a key mechanism to promote Z-decay (10,16,22). In particular, the majority of them (2451 out of 3014; 81.3%) were also targeted by TUT4/7 (Figure 3E; GSE128283 (10)). To confirm the effects of *Pabpn1* depletion on mRNA turnover, several well-characterized Z-decay mRNAs were measured by RT-qPCR, and all of them showed increased stability after *Pabpn1* depletion without affecting the *Tut4/7* mRNA level (Figure 3F and Supplementary Figure S2A). Moreover, the transcripts of several key Z-decay regulators (*Btg4*, *Cnot7*, and *Dis3l2* (see below)) were found to be accumulated after *Pabpn1* depletion (Supplementary Figure S2B), suggesting that PABPN1 also regulates their mRNA decay, which is consistent with previous reports of *Btg4* and *Cnot7* being Z-decay mRNAs (10).

Blocking the Z-decay pathway by depleting the TUT4/7 or BTG4–CCR4–NOT complex causes ZGA failure (10). Thus, we evaluated these downregulated transcripts in *Pabpn1*-depleted 2-cell embryos by gene set enrichment analysis and identified 1317 out of 1998 (65.9%) genes as major ZGA genes (Figure 3G). Over four-fifth of these transcripts (1072; 81.4%) overlapped with the transcripts whose levels were reduced upon *Tut4/7* depletion (Figure 3G; GSE128283 (10)). By gene ontology (GO) analysis, we found that these overlapped zygotic transcripts were involved in ribosome biogenesis and blastocyst development (Supplementary Figure S2C; Table S6), which were in line with the disrupted blastocyst formation in *Pabpn1*-depleted and *Tut4/7*-depleted embryos. Four representative zygotic mRNAs that failed to be expressed in *Pabpn1*-depleted embryos at the 2-cell stage were verified by RT-qPCR (Figure 3H). Taken together, these results indicated that PABPN1-mediated Z-decay is essential for major ZGA in mouse embryos.

### Oligo(U) exonuclease DIS3L2 is required for Z-decay in mouse early embryos

**Figure 4.**
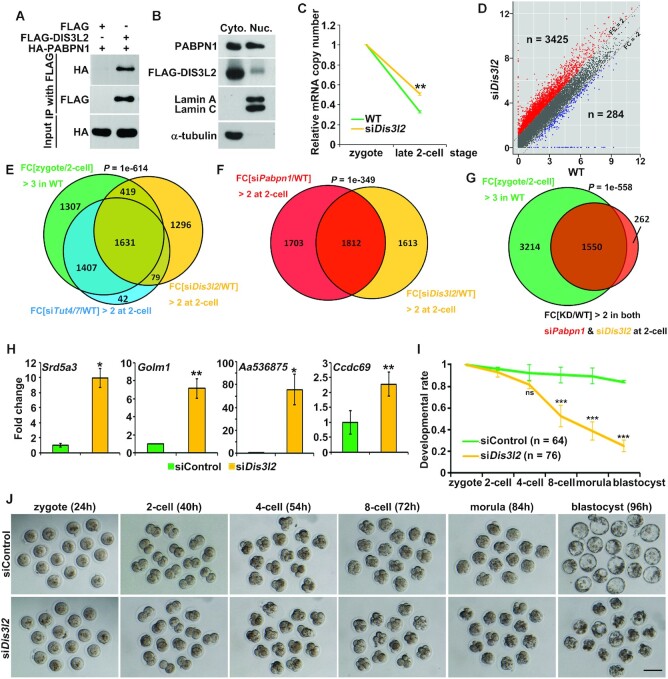
DIS3L2 mediates Z-decay in mouse early embryos. (**A**) Co-immunoprecipitation (Co-IP) experiments showing interactions of PABPN1 with DIS3L2. HeLa cells were transiently transfected with plasmids expressing the indicated proteins for 48 h before lysing and immunoprecipitating with an anti-FLAG affinity gel. Input cell lysates and precipitates were immunoblotted with antibodies against HA. (**B**) HeLa cells were transiently transfected with plasmids expressing FLAG-tagged DIS3L2 and then separated for the cytoplasmic (Cyto.) and nuclear (Nuc.) fractions, which were then subjected to immunoblot of endogenous PABPN1 and FLAG. Lamin A/C and α-tubulin were used as markers of nuclei and cytoplasm proteins, respectively. (**C**) Changes in relative mRNA copy numbers in control and *Dis3l2*-depleted 2-cell embryos. Total mRNA copy numbers were calculated by normalizing with the ERCC spike-in, and mRNA expression at the zygote stage was set to 1.0. **P< 0.01 by two-tailed Student's *t*-test. (**D**) Scatter plot showing the changes in transcript levels in 2-cell embryos after *Dis3l2* depletion. Transcripts that increased or decreased more than 2-fold in *Dis3l2*-depleted embryos are highlighted in red or blue, respectively. (**E**) Venn diagrams showing the overlap of upregulated transcripts in *Dis3l2*-depleted embryos (FPKM (si*Dis3l2*/WT) > 2 in 2-cell embryos) and the degraded transcripts from the zygote to 2-cell embryos in WT (FPKM (zygote/2-cell) > 3 in WT), as well as the upregulated transcripts in *Tut4/7*-depleted embryos (FPKM (si*Tut4/7*/WT) > 2 in 2-cell embryos). *P*= 1e-614 by two-tailed Student's *t*-test. (**F**) Venn diagrams showing the overlap in transcripts whose levels were stabilized at the 2-cell stage of *Pabpn1*-depleted and *Dis3l2*-depleted embryos (FPKM (knockdown/WT) > 2). *P*= 1e–349 by two-tailed Student's *t*-test. (**G**) Venn diagrams showing the overlap of transcripts that are commonly targeted by PABPN1 and DIS3L2 (FPKM (knockdown/WT) > 2 in 2-cell embryos) and the degraded transcripts from the zygote to 2-cell embryos in WT (FPKM (zygote/2-cell) > 3 in WT). *P*= 1e-558 by two-tailed Student's *t*-test. (**H**) RT-qPCR results showing the relative mRNA levels of indicated Z-decay transcripts in 2-cell embryos with or without *Dis3l2* depletion. Error bars, S.E.M. *P< 0.05, **P< 0.01 by two-tailed Student's *t*-test. *n* = 3 biological replicates. (**I**) Zygotes collected from oviducts were microinjected with si*Dis3l2* and then cultured for 96 h. Developmental rates of embryos at the indicated stages were evaluated. The number of analyzed embryos was indicated (n). Error bars, S.E.M. ***P< 0.001 by two-tailed Student's *t*-test. ns: non-significant. (**J**) Representative images of embryos after treatments with si*Dis3l2* and control embryos that developed to the corresponding stages. Scale bar = 100 μm.

To understand the roles of PABPN1 in the Z-decay pathway, we focused on DIS3L2, an exonuclease that specifically degrades oligouridylated RNA. We first determined the PABPN1-DIS3L2 cooperation by transiently transfecting plasmids expressing HA-tagged PABPN1 and FLAG-tagged DIS3L2 into HeLa cells, and co-immunoprecipitation (Co-IP) showed that PABPN1 interacts with DIS3L2 (Figure 4A). DIS3L2 is a cytoplasmic factor (30,31), whereas PABPN1 is well known for its nuclear functions. Therefore, we performed subcellular fractionation measurements of PABPN1 in HeLa cells. WB analysis of proteins isolated from nuclear and cytoplasmic fractions revealed PABPN1 and DIS3L2 co-accumulation in the cytoplasm (Figure 4B), consistent with the cytoplasmic distribution of PABPN1 in mouse early embryos (Figure 1B, C). Therefore, DIS3L2 is a potential cytoplasmic PABPN1 partner.

Next, we investigated the functions of DIS3L2 during the mouse Z-decay process (19,32). *Dis3l2* expression in oocytes and early embryos was characterized by RT-qPCR. *Dis3l2* was highly expressed in the GV stage, yet the transcripts were significantly eliminated during the MZT while zygotic *Dis3l2* was activated at the 4-cell stage (Supplementary Figure S3A). We failed to detect DIS3L2 protein during the mouse MZT due to the low efficiency of the commercially available DIS3L2 antibody (data not shown). However, both the ribosome-seq data in zebrafish and proteomic data in *Xenopus* previously revealed that *Dis3l2* was actively translated from maternally deposited mRNAs during the MZT (16,33), implying the underlying requirement of DIS3L2 for the MZT process. To further explore *Dis3l2* function during mouse early embryogenesis, we depleted *Dis3l2* from the zygote stage by siRNA microinjection with verified high knockdown efficiency (Supplementary Figure S3B) and subjected *Dis3l2*-depleted late 2-cell stage embryos to RNA-seq. Gene transcript level analysis showed that total mRNA levels were increased at the late 2-cell stage when *Dis3l2* was depleted (Figure 4C), and specifically, 3425 transcripts were upregulated, and only 284 transcripts were downregulated as compared to the control (Figure 4D). Gene set enrichment analysis of the upregulated transcripts suggested that 1631 (out of 3425; 47.6%) transcripts overlapped with published TUT4/7-targeted Z-decay transcripts (Figure 4E; GSE128283 (10)), implying a contribution of DIS3L2 to the TUT4/7-mediated Z-decay process. In addition, there was considerable overlap (1,812) between the transcripts that accumulated in *Pabpn1*-depleted (3515; overlap ratio: 51.6%) and *Dis3l2*-depleted (3425; overlap ratio: 52.9%) 2-cell embryos (Figure 4F; GSE128283 (10)); among these, 1550 of the 1812 (85.5%) co-regulated transcripts were those being degraded in WT 2-cell embryos (Figure 4G). RT-qPCR results showed that representative Z-decay transcripts commonly targeted by PABPN1 and DIS3L2 based on RNA-seq results were remarkably enriched at the 2-cell stage after *Dis3l2* depletion (Figure 4H).

mRNA 3′-ligation RACE of well-characterized Z-decay mRNA *Gm4745* mRNA at the 2-cell stage was performed to visualize the accumulation of 3′-oligouridylates (Supplementary Figure S3C) (10). In brief, mRNAs derived from *Pabpn1*- or *Dis3l2*-depleted 2-cell embryos were appended with 3′-linker, followed by *Gm4745* tails amplification using RT-PCR (Supplementary Figure S3D), and further sequenced after TA cloning. 3′-Oligouridylation of *Gm4745* was detected in a wide range of poly(A) tail lengths in control embryos. However, a pronounced enrichment of 3′-oligouridylation among short A tails (<20 nt) was identified after *Pabpn1* or *Dis3l2* depletion. (Supplementary Figure S3E). Taken together, these results suggested that DIS3L2 removes 3′-oligouridylated mRNAs during mouse Z-decay in a complementary manner with PABPN1, downstream of the TUT4/7-induced uridylation step.

To understand the physiological function of DIS3L2-mediated Z-decay, we examined the developmental phenotype. *Dis3l2* depletion diminished embryonic development after the 4-cell stage, and *Dis3l2*-depleted embryos failed to develop into blastocysts (Figure 4I-J), presenting a developmental arrest comparable to that in *Tut4/7*-depleted embryos (10). This indicates that Z-decay is critically required for preimplantation-stage embryonic development and that disrupting any step across the Z-decay pathway will arrest embryonic development.

### PABPN1 promotes Z-decay by recruiting DIS3L2 to Z-decay transcripts in the cytoplasm

**Figure 5.**
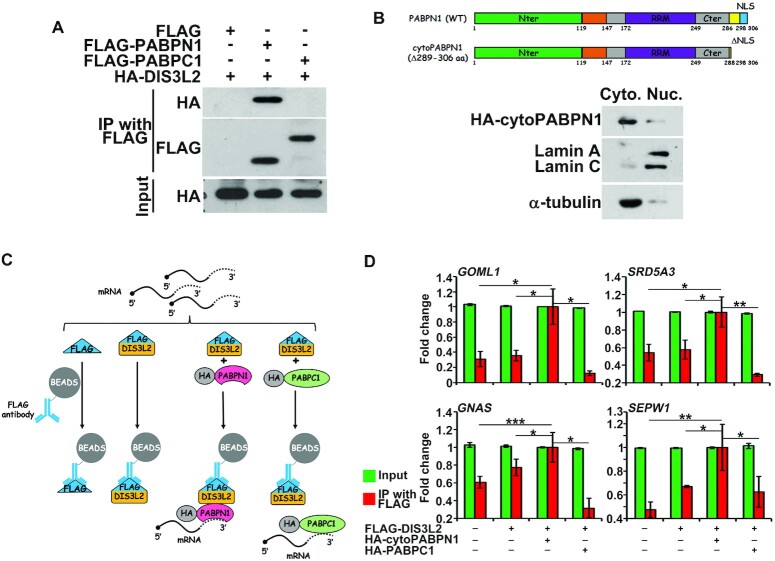
PABPN1 increases the RNA-binding affinity of DIS3L2 involved in Z-decay. (**A**) Co-IP experiments showing that PABPN1, but not PABPC1, binds to DIS3L2. HeLa cells transiently transfected with plasmids encoding the indicated proteins were lysed and subjected to IP with an anti-FLAG affinity gel. Input cell lysates and precipitates were immunoblotted with antibodies against HA. (**B**) Subcellular fractionation showing the distribution of cytoPABPN1, a PABPN1 lacking the nuclear localization signal. HeLa cells were transfected with plasmids expressing HA-tagged cytoPABPN1 and then separated for the cytoplasmic (Cyto.) and nuclear (Nuc.) fractions, the subjected to immunoblot for HA. Lamin A/C and α-tubulin were used as markers of nuclei and cytoplasm proteins, respectively. (**C**) Schematic description of RNA immunoprecipitation (RIP) procedure. (**D**) RIP results showing the interaction of DIS3L2 with the indicated transcripts, with or without cytoPABPN1/PABPC1 overexpression. HeLa cells were co-transfected with plasmids expressing FLAG-tagged DIS3L2 and HA-tagged cytoPABPN1/PABPC1 for 48 h before immunoprecipitation. RNAs recovered from the immunoprecipitants were subjected to RT-qPCR. Relative changes in values of both input and IP samples were normalized by the values obtained from RIP for the FLAG-DIS3L2 and HA-cytoPABPN1 co-expression group. Error bars, S.E.M. The *P*-value represents the two-tailed Student's *t*-test comparing the RIP results of DIS3L2 with the indicated transcripts in the presence of PABPN1, *P< 0.05, **P< 0.01, and ***P< 0.001 by two-tailed Student's *t*-test. *n* = 3 biological replicates.

The fact that the cytosolic PABPN1 collaborated with DIS3L2 in mediating 3′-oligouridylated mRNAs degradation during Z-decay prompts the question of whether the cytoplasmic function of PABPN1 can be substituted by a similar cytoplasmic PABP, such as PABPC1, the most abundant paralog of the PABPC family. Thus, we performed Co-IP to detect the interaction between PABPC1 and DIS3L2. The results showed a PABPN1-DIS3L2 interaction in the positive control; however, PABPC1 did not interact with DIS3L2 (Figure 5A).

We hypothesized that the unique cytoplasmic PABPN1-DIS3L2 binding tethers DIS3L2 to mRNAs during Z-decay. To eliminate the possibility of nuclear PABPN1 interference, we deleted the last 18 aa of WT PABPN1 (^289^RGRVYRGRARATSWYSPY^306^) (34) and obtained a PABPN1 form that localized entirely within the cytoplasm, termed cytoPABPN1 (Figure 5B). DIS3L2-RNA immunoprecipitation (RIP) assays were performed in the presence and absence of cytoPABPN1. The results showed that cytoPABPN1 effectively enhanced binding between DIS3L2 and representative Z-decay transcripts (Figure 5C, D). Conversely, PABPC1 did not increase the mRNA-binding ability of DIS3L2 (Figure 5C, D). These results suggested that PABPN1 strengthens the binding between DIS3L2 and mRNAs in the cytoplasm during the Z-decay process. Taken together, the results suggested that TUT4/7 labeled the Z-decay transcripts with terminal uridylation, and then PABPN1 recruited DIS3L2 to the Z-decay transcripts to promote Z-decay in the cytoplasm.

### PABPN1 is essential for embryonic genome activation and preimplantation embryo development

**Figure 6.**
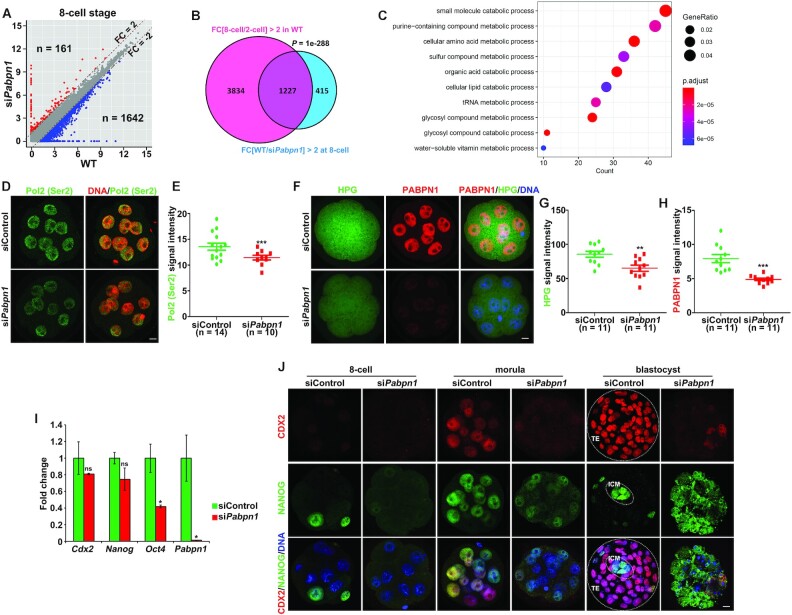
PABPN1 is essential for embryonic genome activation and preimplantation embryo development. (**A**) Scatter plot showing the level changes of transcripts in 8-cell embryos after *Pabpn1* depletion. Transcripts increased or decreased more than 2-fold in *Pabpn1*-depleted embryos are highlighted in red or blue, respectively. (**B**) Venn diagram showing the overlap of transcripts that were upregulated during the 2-to-8-cell transition in WT (FPKM (8-cell/2-cell) > 2) and those that were downregulated in *Pabpn1*-depleted embryos compared to WT at the 8-cell stage (FPKM (WT/si*Pabpn1*) > 2 in 8-cell embryos). *P*= 1e–288 by two-tailed Student's t-test. (**C**) Functional categorization of the embryonic transcripts downregulated (> 2 folds) in *Pabpn1*-depleted 8-cell embryos (indicated by the overlapped transcripts in (B)) by GO analysis. (**D** Immunofluorescence results of the phosphorylated RNA polymerase II CTD repeat YSPTSPS (Pol2 [Ser2]) (green) in WT and *Pabpn1*-depleted 8-cell embryos. Nuclei were labeled by DAPI (red). Scale bar = 10 μm. (**E**) Quantification of Pol2 (Ser2) signals in (D). The numbers of analyzed embryos are indicated (*n*). Error bars, S.E.M. ***P< 0.001 by two-tailed Student's *t*-test. (**F**) L-HPG (a methionine analog) fluorescent (green) staining shows protein synthesis activity in WT and *Pabpn1*-depleted 8-cell embryos. PABPN1 (red) was co-stained to indicate knockdown efficiency. Scale bar = 10 μm. Quantification of the HPG (**G**) and PABPN1 (**H**) signals are shown in (**F**). The numbers of analyzed embryos are indicated (n). Error bars, S.E.M. **P< 0.01, ***P< 0.001 by two-tailed Student's *t*-test. (**I**) RT-qPCR results show the relative mRNA levels of indicated transcripts in 8-cell embryos with or without *Pabpn1* depletion. Error bars, S.E.M. *P< 0.05 by two-tailed Student's *t*-test. ns: non-significant. *n* = 3 biological replicates. (**J**) Immunofluorescence for CDX2 and NANOG in control and *Pabpn1*-depleted embryos at the time point when the control embryos have developed to the 8-cell, morula, and blastocyst stages. *n* = 10 embryos at each stage. Scale bar = 10 μm.

Based on the finding that *Pabpn1*-depleted embryo development was completely blocked before the blastocyst stage, a more drastic defect than *Tut4/7* depletion caused a lower blastocyst developmental rate (10); we further investigated the potential impact of PABPN1 on the transcriptional activity after major ZGA. The embryonic genome activation involves periodic transcriptional activities, including two major waves: the first phase is a major ZGA at the late 2-cell stage; the second phase initiates at the 4- to 8-cell transition (1). Gene expression analysis revealed that *Pabpn1* depletion resulted in a higher number of downregulated than upregulated transcripts in 8-cell embryos (1,642 versus 161) (Figure 6A). Gene set enrichment analysis of the downregulated transcripts suggests that 74.7% (1,227 out of 1,642) of these transcripts were expressed during the 4- to 8-cell transition in WT embryos (Figure 6B). Interestingly, *Pabpn1*-affected embryonic transcripts were enriched in various metabolic processes (Figure 6C; Supplementary Table S6). Global transcription activity of the second wave of ZGA was also analyzed by phosphorylated RNA polymerase II C-terminal domain (CTD) repeat YSPTSPS (Pol2 [Ser2]), also a marker of transcription activation) (9,35); Pol2 (Ser2) signals were expressed in the nuclei of the tested embryos but weakened after *Pabpn1* depletion (Figure 6D, E), indicating that *Pabpn1* depletion impairs the embryonic genome transcription during the 4- to 8-cell transition. These results combined with the previous ones showing that PABPN1 is essential for major ZGA led to the conclusion that zygotic *Pabpn1* is required for the progression of successful embryonic genome transcription during early embryogenesis.

At the 8-cell stage, embryos tend to express proteins that are involved in protein translation, according to the proteomic data (36). HPG (a methionine analog) incorporation assay was utilized to evaluate the global level of protein synthesis (37). The depletion of *Pabpn1* induced weaker HPG signals than those in the control group, indicating that total protein synthesis decreased after *Pabpn1* depletion (Figure 6F–H).

Blastocysts undergo primary differentiation leading to the delineation of the trophectoderm (TE) and the inner cell mass (ICM). *Cdx2*, *Nano*g and *Oct4* are essential for cell fate commitment (38). Since *Pabpn1*-depleted embryos failed to develop into blastocysts, we further examined the effects of PABPN1 on the expression of cell fate-determining factors. RT-qPCR was performed to determine the mRNA levels of *Cdx2*, *Nanog* and *Oct4* in WT and *Pabpn1*-knockout 8-cell embryos. *Oct4* was slightly downregulated after *Pabpn1* depletion (Figure 6I). Immunofluorescence assay results revealed that CDX2 protein levels significantly decreased in *Pabpn1*-knockout embryos (Figure 6J). These results suggested that PABPN1 is required for the expression of cell fate-determining factors in preimplantation embryos. In addition, the *Dis3l2*-knockout embryos also failed to develop into blastocysts and displayed a disrupted accumulation of CDX2 and NANOG proteins in preimplantation embryos (Supplementary Figure S4A). Collectively, the PABPN1- and DIS3L2- regulated Z-decay process is required to express embryonic factors that are essential for early embryo development, especially for cell fate determination.

## DISCUSSION

During the MZT, the clearance of maternally encoded mRNAs is a key event, which has been studied in model systems and reportedly consists of two combined activities: M-decay and Z-decay (4,10). Small RNAs, mainly microRNAs, were previously identified as facilitators of Z-decay in *Drosophila*, zebrafish, and *Xenopus* (39–41). However, in mice, the microRNAs are functionally inactive, and there is still no direct evidence that zygotic microRNAs play a role in maternal mRNA clearance (42,43). Recent studies revealed that zygotically expressed TUT4 and TUT7 and their mRNA 3′-oligouridylation are required for mediating mouse Z-decay (10,16,22). While TUT4/7 alone is not sufficient to accomplish maternal mRNA decay, the factors that are actively expressed during zygotic genome activation (ZGA) that drive these processes remain unidentified. Our study identified PABPN1 as a pivotal zygotic factor that facilitates Z-decay during the mammalian MZT. PABPN1 facilitates binding of DIS3L2 exonuclease to 3′-oligouridylated mRNAs, downstream of TUT4/7-mediated 3′-oligouridylation, promoting maternal mRNA degradation post-fertilization.

*Drosophila pabpn1-*ortholog *pabp2* mutants showed early developmental arrest due to extended poly(A) tails in specific mRNAs, which affected their encoded protein levels (44). Even though the mechanism by which *Drosophila* PABP2 controls mRNA turnover remains poorly understood, it is known that PABP2 plays an essential role during early development. In this study, *Pabpn1*-knockout mouse embryos were shown to be arrested and die at the morula stage. In addition, the *Pabpn1* gene was transcriptionally activated post-fertilization, and its protein levels were detected and promptly increased during embryogenesis. Hence, *Pabpn1* was identified as a novel zygotic factor required for mouse early embryonic development.

PABPN1 was previously known as a multi-functional mediator of nuclear polyadenylation, involved in (i) harboring on the growing poly(A) tail and interacting with poly(A) polymerase (45); (ii) controlling poly(A) tail length (46); (iii) modulating alternative cleavage and polyadenylation (47). Although PABPN1 is defined as a nuclear poly(A)-binding protein, it appears cytoplasmically in the peri-nuclear region, suggesting that it has a role as a shuttle between the nucleus and cytoplasm (48). However, PABPN1 functions outside of the nucleus are still unclear. This study revealed an unforeseen cytoplasmic function of PABPN1 coupled with early embryonic development. Several lines of evidence included: (i) the outline of the unique nuclear-cytoplasmic pattern of PABPN1 distribution during early embryonic development in mice, noticing that the cytoplasmic localization overlapped with the window of Z-decay process; (ii) the observation of substantial overlap between PABPN1-targeted transcripts and Z-decay mRNAs; (iii) the observation that PABPN1 interacted with the cytoplasmic exonuclease DIS3L2 and cytosolic mRNAs in Co-IP and RIP assays, respectively. Moreover, these findings are consistent with the identification of a cytoplasmic PABPN1 found in *Drosophila* and *Xenopus* embryos (44,49).

This experimental evidence could be challenged by a controversy about whether the cytoplasmic function of PABPN1 can be replaced by cytoplasmic steady-state PABPC1. However, in our experiments, PABPC1 did not interact with DIS3L2 in Co-IP assays; also, PABPC1 did not increase DIS3L2-mRNA-binding affinity, as demonstrated by RIP assay. Thereby, we concluded that cytoplasmic PABPN1 is indispensable for Z-decay. Recent studies further support the presence of a cytoplasmic role for PABPN1 during Z-decay. The footprint of an individual monomer of PABPC1 is 27 nt *in vivo*, exceeding the length of the poly(A)-tail targeted by TUT4/7 (<25 nt), suggesting PABPC1 protects polyadenylated mRNAs against TUT4/7 (19,24). Conversely, PABPN1 recognizes 10–11 nucleotides (50), which seems reasonable for a platform covering the short poly(A)-tail of 3′-oligouridylated mRNAs, promoting their degradation. Therefore, PABPC1 and cytoplasmic PABPN1 play different roles in regulating mRNA stability.

The Z-decay pathway has been recently demonstrated to be physiologically essential for mammalian early embryo development (10,11). For instance, if BTG4-CCR4-NOT-mediated deadenylation or TUT4/7-mediated oligouridylation is blocked, early embryogenesis will fail (10). This study also confirms the conclusion that the disruption of the PABPN1-mediated oligouridylated mRNA decay is lethal to embryos at the morula stage. Further analysis revealed that the deficiency of these key factors involved in Z-decay regulation causes ZGA defect, with common global zygotic transcript downregulation at the 2-cell stage. *Pabpn1*-knockout embryos failed to develop into blastocysts, a more severe developmental defect than embryos derived from BTG4 trimmed-away or *Tut4/7* depletion. Moreover, PABPN1 supports further embryonic genome activation during the 4-to-8-cell transition, corresponding to most of PABPN1 transported back into the nuclei. Due to the multiple nuclear roles of PABPN1 in pre-mRNA processing and 3′-end polyadenylation, *Pabpn1* depletion has a general effect on mRNA biosynthesis (45–47). The *de novo* synthesized zygotic transcripts are likely to be defective and degraded, which could be a reason for the severe phenotype in addition to Z-decay defects.

We have previously shown that PABPN1L, a maternally expressed PABPN1 homolog, acts as a poly(A) adaptor to recruit BTG4 and CCR4–NOT deadenylase to mediate maternal mRNA clearance (15). Both nuclear PABPs are present in the cytoplasm and facilitate maternal mRNA decay during the MZT, but function in a divergent way: (i) *Pabpn1l*^−/−^ mice are viable and healthy, but females are infertile owing to early developmental arrest, at the 1- to 2-cell stage, of the resultant embryos, which differs from the observations of *Pabpn1* knockout that caused morula stage lethality; (ii) in agreement with these phenotypes, the expression window of *Pabpn1l* is temporally separated from the expression pattern of *Pabpn1* during the MZT and early embryogenesis; PABPN1L is maternal deposited and its expression is restricted before the 2-cell stage, whereas *Pabpn1* is significantly activated during ZGA; (iii) PABPN1L is an mRNA-binding adapter of BTG4 that participates in M-decay; in contrast, PABPN1 enhances DIS3L2-mRNA interactions, promoting Z-decay transcript degradation. Taken together, PABPN1L and PABPN1 function successively guarantee that maternal mRNA is degraded in a timely fashion, which is a prerequisite for early embryonic development.

PABPN1 is ubiquitously expressed and is emerging as a major regulator in alternative cleavage and polyadenylation (APA) (47,51). The dominant mutations linked to alanine-expanded PABPN1 form nuclear aggregates which sequester normal PABPN1, and enhance the usage of proximal cleavage sites, leading to oculopharyngeal muscular dystrophy (OPMD) (47,51,52). A *Drosophila* model of OPMD showed that alanine-expanded PABPN1 increases cytoplasmic amounts of PABPN1 (53). These findings clarified the mechanisms of PABPN1 in modulating cytoplasmic mRNA stability during mouse early development, providing insights into the therapeutic potential of OPMD.

**Figure 7.**
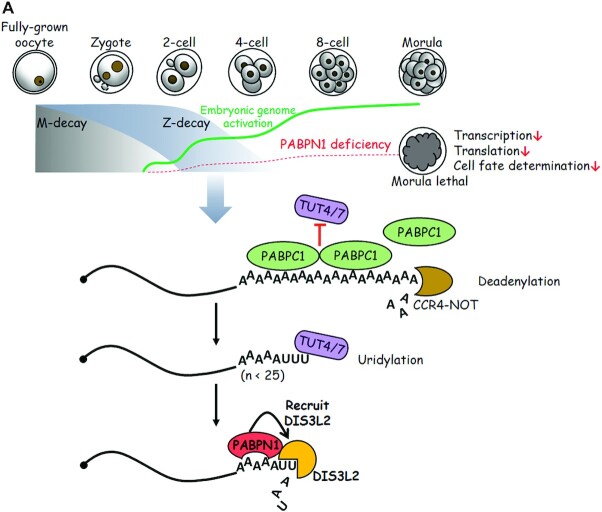
Schematic diagram showing PABPN1 as a zygotic factor that mediates Z-decay. (**A**) Maternal mRNAs are removed by two pathways: M-decay (gray shadow) and Z-decay (blue shadow). Relatively short-lived maternal transcripts are degraded by M-decay, which is exclusively mediated by maternal factors during oocyte maturation and fertilization. The remained transcripts are depleted after ZGA via Z-decay. Z-decay transcripts are deadenylated by the CCR4-NOT complex, and PABPC1 is isolated from mRNAs as poly(A) tails get shorter. TUT4/7 attend to uridylate PABPC1-free mRNAs with short poly(A)-tails (less than ∼25 nt). *Pabpn1* is expressed during ZGA, acts on TUT4/7-mediated 3′-oligouridylated mRNAs, and recruits exonuclease DIS3L2 to facilitate their decay. Maternally supplied mRNA clearance guarantees the establishment of preimplantation developmental competence. In the absence of PABPN1, the embryos fail to gain developmental competence (included transcription, translation, cell fate determination), and embryogenesis is blocked at the morula stage. Green and red dashed curves represent the expression level of embryonic transcripts in WT and *Pabpn1*-depleted embryos, respectively.

In summary, cytoplasmic PABPN1 binds poly(A) tails of 3′-oligouridylated mRNAs during early embryonic stages and removes these maternally deposited mRNAs recruiting exonuclease DIS3L2 (modeled in Figure 7). This study uncovered novel cytoplasmic functions and physiological significance of PABPN1 and broadened the horizon of the Z-decay pathway in mammals.

## DATA AVAILABILITY

RNA-seq data have been deposited in the NCBI Gene Expression Omnibus database. GEO accession number: GSE174032. RNA-seq data for *Tut4/7* was deposited previously (10); the GEO accession number is GSE128283.

## Supplementary Material

gkab1213_Supplemental_Files
