# Supplementary material for: Nuclear poly(A) binding protein 1 (PABPN1) mediates zygotic genome activation-dependent maternal mRNA clearance during mouse early embryonic development
Source: Nucleic Acids Res. 2021 Dec 14;50(1):458–72. doi: 10.1093/nar/gkab1213 (PMC8855302; doi:10.1093/nar/gkab1213)
Supplement: gkab1213_Supplemental_Files [file gkab1213_Supplemental_Files.zip › Pabpn1_Z-decay_Sup File_R1.pdf]

Supplementary Figures

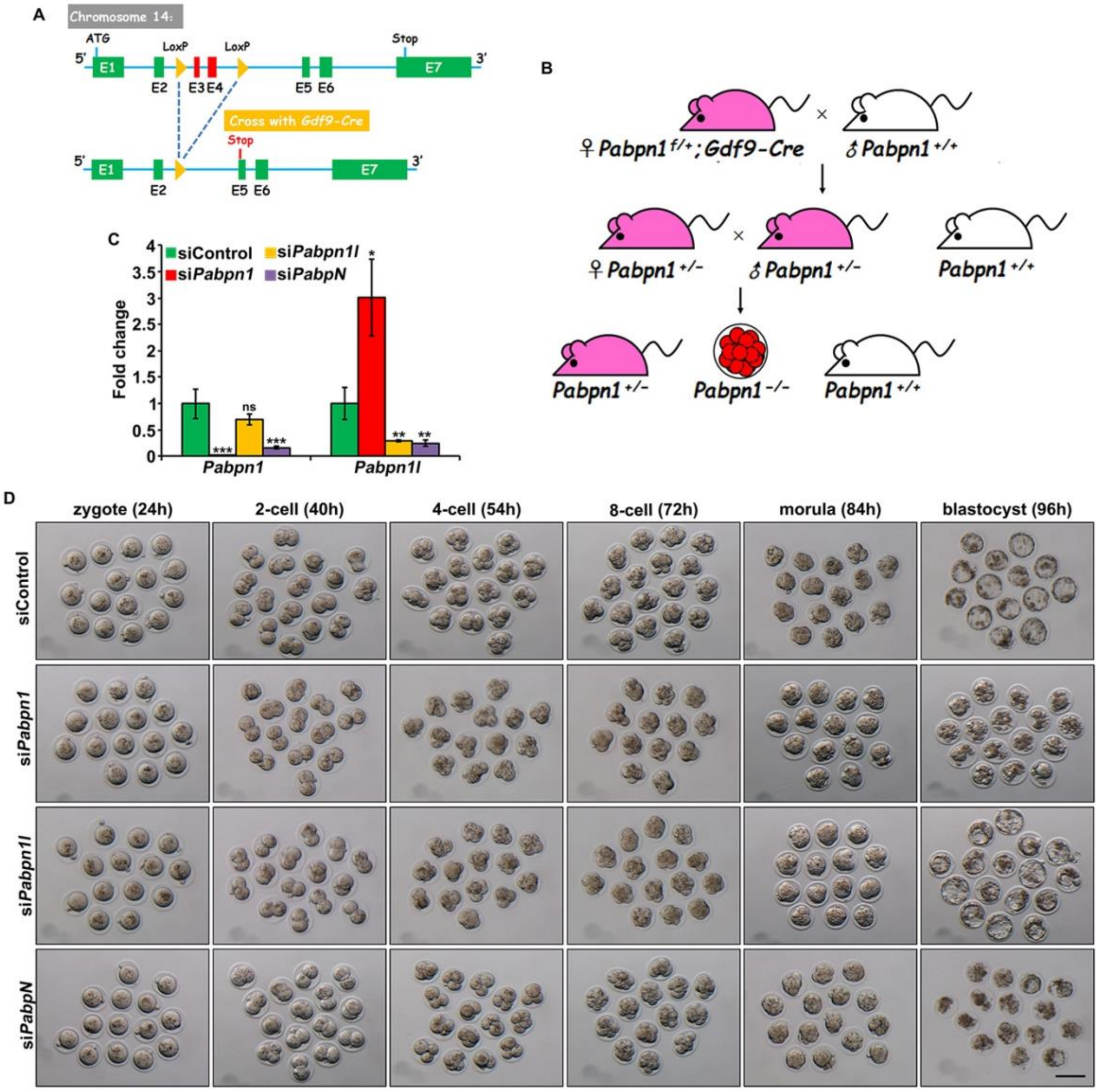

Supplementary Figure S1: *Pabpn1* depletion causes preimplantation mortality in mice. A. Schematic illustration of the insertion of loxP sequences into the mouse *Pabpn1* gene locus and the generation of *Pabpn1*-null allele. Stop, stop codon; E1-E7, exon1-7. **B.** Schematic diagram showing the mating strategies used to obtain *Pabpn1*-knockout mice. **C.** RT-qPCR results showing the relative mRNA levels of *Pabpn1* or *Pabpn1l* in 2-cell embryos following the indicated treatments. Error bars, S.E.M. *$p < 0.05$, **$p < 0.01$, and ***$p < 0.001$ by two-tailed Student's *t*-test. ns: non-significant. n = 3 biological replicates. **D.** Representative images of the embryos after the treatments are shown in Figure 2C when control embryos have developed to the corresponding stages. Scale bar = 100 μm.

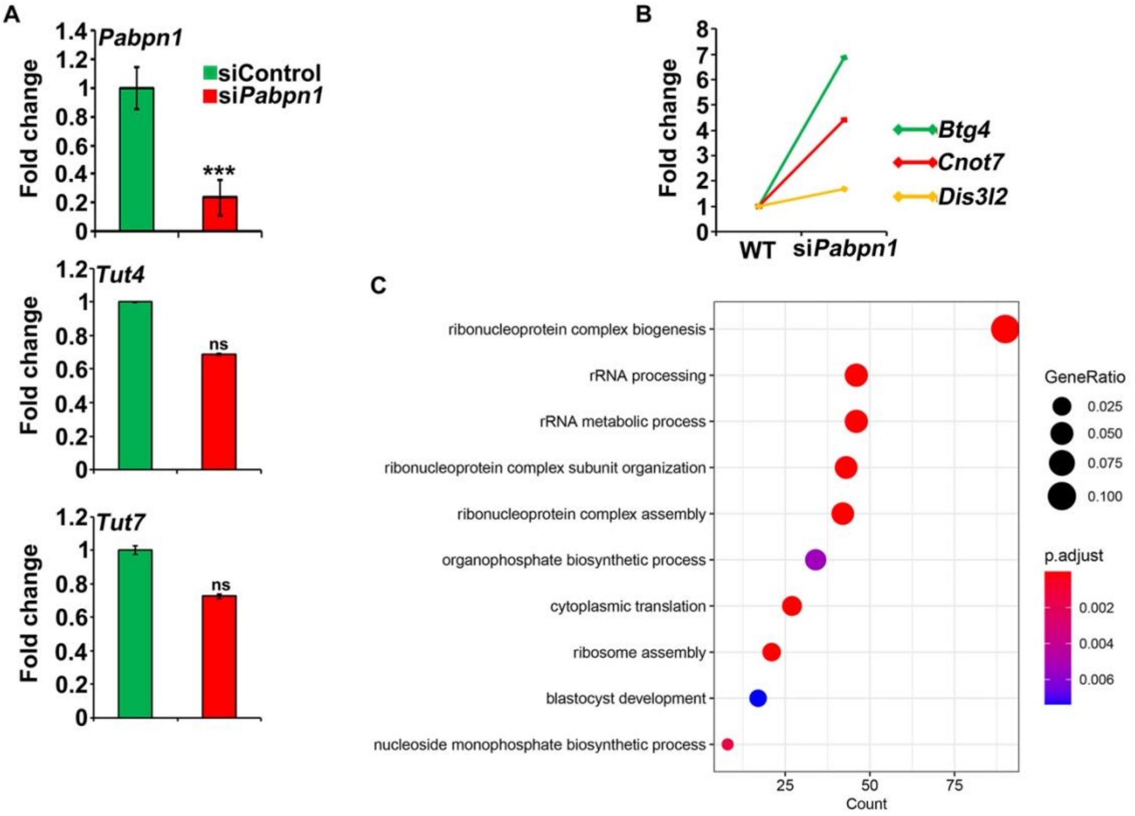

Supplementary Figure S2: PABPN1 is required for Z-decay and zygotic genome activation (ZGA). A. RT-qPCR results showing mRNA levels of *Pabpn1* and *Tut4/7* in *Pabpn1* depleted 2-cell embryos. Small interfering RNAs (siRNAs) were microinjected into zygotes at 24 h after hCG injection. Error bars, S.E.M. ***$p < 0.001$ by two-tailed Student's *t*-test. ns: non-significant. n = 3 biological replicates. **B.** Changes in mRNA level of *Btg4*, *Cnot7*, and *Dis3l2* at the 2-cell stage upon *Pabpn1* knockdown. The indicated mRNA level in WT embryos was set to 1.0 for analysis. **C.** Functional categorization of the zygotic transcripts downregulated (> 2 folds) in both *Pabpn1*-depleted and *Tut4/7*-depleted 2-cell embryos (indicated by the overlapped transcripts in Figure 3G) by gene ontology analysis.

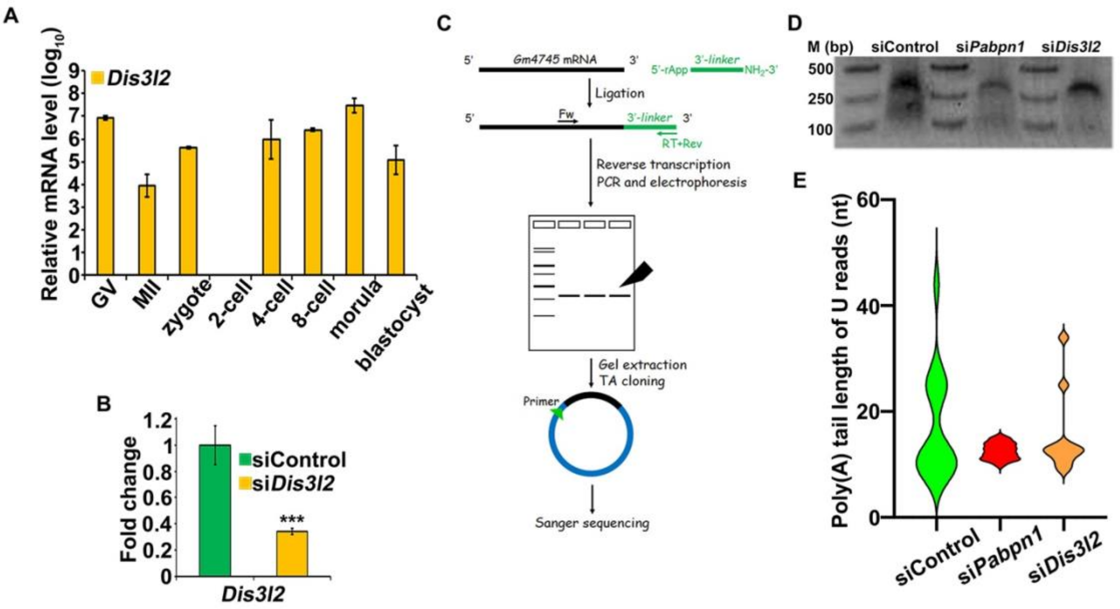

Supplementary Figure S3: DIS3L2 remove 3'-oligouridylated mRNAs during mouse Z-decay. A. RT-qPCR results showing relative expression levels of mouse *Dis3l2* in oocytes and preimplantation embryos. Error bars, S.E.M. n = 3 biological replicates. The relative mRNA level in 2-cell embryos was set to 1.0, and $\log_{10}$ fold changes at different stages were shown. **B.** RT-qPCR results showing the RNAi depletion efficiency of *Dis3l2* in GV oocytes. siRNAs were microinjected into oocytes at 44 h after PMSG injection. Error bars, S.E.M. ***$p < 0.001$ by two-tailed Student's *t*-test. n = 3 biological replicates. **C.** Schematic description of the *Gm4745* 3'-ligation RACE experimental procedure. *Gm4745* mRNA was ligated to 3′-linker and reverse transcribed, and its mRNA tails were amplified with *Gm4745*-specific forward primer and RT+reverse primer (Supplementary Table S1). PCR products were visualized on 2% agarose gels (**D**), then extracted and cloned into T-vectors for Sanger DNA sequencing. **D.** 3′-ligation RACE PCR products of *Gm4745* transcripts in WT, *Pabpn1*-depleted, and *Dis3l2*-depleted 2-cell embryos were resolved by agarose gel. **E.** Violin plot analysis comparing the distributions of 3′-oligouridylation in *Gm4745* transcript poly(A) tail derived from WT, *Pabpn1*-depleted, and *Dis3l2*-depleted 2-cell embryos. The vertical position represents the length of the *Gm4745* transcript poly(A) tail.

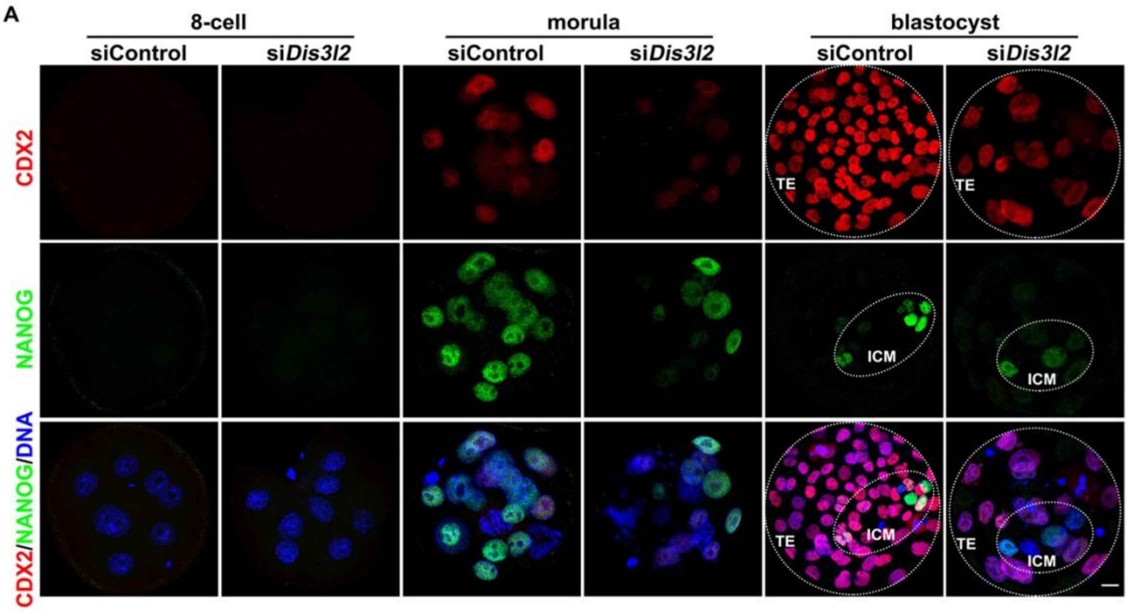

Supplementary Figure S4: *Dis3l2* deficiency disrupts the establishment of preimplantation developmental competence. A. Immunofluorescence of CDX2 and NANOG in control and *Dis3l2*-depleted embryos at the time point when the control embryos have developed to the 8-cell, morula, and blastocyst stages. n = 10 embryos at each stage. Scale bar = 10 μm.

Supplementary Tables

Supplementary Table S1. Primer sequences.

Primer name	Genes targeted	Application	Sequences (5’-3’)
sgRNA-up1	m*Pabpn1*	CRISPR-CAS9 mediated mutations	5’-TAGGAACTGACTGGTGGCTGTA-3’
sgRNA-down1			5’-AAACTACAGCCACCAGTCAGTT-3’
sgRNA-up2	m*Pabpn1*		5’-TAGGCCTAACTTGAGCTGGGCG-3’
sgRNA-down2			5’-AAACCGCCCAGCTCAAGTTAGG-3’
GT-F	m*Pabpn1*	Genotyping WT KO	5’-GATAGTCTTGTGCCTTCCTTTGTCTC-3’
GT-R (WT)			5’-TCGAGTCTCTATTCAGCTTTAGCTGAAA-3’
GT-R (KO)			5’-CAGTCTTTAGTTTATTACCTGGTCTCTGAAG-3’
m*Pabpn1*-F	m*Pabpn1*	Real-time PCR	5’-GGTGATTCCCAAACGAACCAACAG-3’
m*Pabpn1*-R			5’-GTCGCTCTAGCCCGGCCC-3’
m*Pabpn1l*-F	m*Pabpn1l*	Real-time PCR	5’-GTACTTCCCAAAAGGACGAACTTCCC-3’
m*Pabpn1l*-R			5’-GGAGAGAACCACTGTGGTGCTCCTC-3’
m*Gm4745*-F	m*Gm4745*	Real-time PCR	5’-TGTGGAGCTGGCTGTATTG-3’
m*Gm4745*-R			5’-GAGGTTCATCTGCCTGTACTTAG-3’
m*Srd5a3*-F	m*Srd5a3*	Real-time PCR	5’-GTCATCCGCCCATCAGTATAAA-3’
m*Srd5a3*-R			5’-GTACTCGAACCAGTCTCCAAAG-3’
m*Golm1*-F	m*Golm1*	Real-time PCR	5’-CCTATGACCTGAACCAGTGTATC-3’
m*Golm1*-R			5’-GCTTTCTGATGACCTCCTCTATC-3’
m*Ccdc69*-F	m*Ccdc69*	Real-time PCR	5’-CCCAAGAAGTGCCTTTCCTTA-3’
m*Ccdc69*-R			5’-GCCACGGGAGAAGTCTTAAAT-3’
m*Aa536875*-F	m*Aa536875*	Real-time PCR	5’-GTGATCTGTCTCTGGCTGAAAG-3’
m*Aa536875*-R			5’-TGAGTGTTCCTGCATCCATTAG-3’
h*SRD5A3*-F	h*SRD5A3*	Real-time PCR	5’-AAATCTATTGATGCAAGCACGGTGGT-3’
h*SRD5A3*-R			5’-GAAACGTAGATCATCAGCTCTGCTAAGTAGTTA-3’
h*GOLM1*-F	h*GOLM1*	Real-time PCR	5’-AGAGGAATTACGGCAGGCTGCA-3’
h*GOLM1*-R			5’-GCTGTCTCTGGTCGTTGTTTTCACTC-3’
h*GNAS*-F	h*GNAS*	Real-time PCR	5’-CTGGAGAATCTGGTAAAAGCACCATTG-3’
h*GNAS*-R			5’-TTCAGGTTGTTTTTGATGTCCTGCAC-3’
h*SEPW1*-F	h*SEPW1*	Real-time PCR	5’-GCCGTCCGAGTCGTTTATTGT-3’
h*SEPW1*-R			5’-CCGGGGAACTCATCTTCTAACT-3’
m*Dux*-F	m*Dux*	Real-time PCR	5’-AGCGACTCAAACTCCTTCTTC-3’
m*Dux*-R			5’-CTGTGCTGTCTGCTTGAGT-3’
m*Npl*-F	m*Npl*	Real-time PCR	5’-GGCCTTCCCTAAGAAGAAACTC-3’
m*Npl*-R			5’-CCTGTTCCTTCACCAGGTAATC-3’

m*Guca1a*-F	m*Guca1a*	Real-time PCR	5’-ACCGAGTGCCATCAGTGGTAT-3’
m*Guca1a*-R			5’-CTGCCACGTACTCCATGAAGT-3’
m*Gata4*-F	m*Gata4*	Real-time PCR	5’-CCCTACCCAGCCTACATGG-3’
m*Gata4*-R			5’-ACATATCGAGATTGGGGTGTCT-3’
m*Cdx2*-F	m*Cdx2*	Real-time PCR	5’-AGCTGCTGTAGGCGGAATGTATG-3’
m*Cdx2*-R			5’-TCAGTGACTCGAACAGCAGCAA-3’
m*Nanog*-F	m*Nanog*	Real-time PCR	5’-TTCTTGCTTACAAGGGTCTGC-3’
m*Nanog*-R			5’-AGAGGAAGGGCGAGGAGA-3’
m*Oct4*-F	m*Oct4*	Real-time PCR	5’-ATGGGGAAAGAAGCTCAGTG-3’
m*Oct4*-R			5’-CAAAATGATGAGTGACAGACAGG-3’
m*Dis3l2*-F	m*Dis3l2*	Real-time PCR	5’-GAGTGCTGTTGGTGCTTCG-3’
m*Dis3l2*-R			5’-GGTTTAACTGCCTTCCACTGAT-3’
Pabpn1-F1	*mPabpn1*	siRNA	5’-GGUUCAGUCAACCGUGUUATT-3’
Pabpn1-R1			5’-UAACACGGUUGACUGAACCTT-3’
Pabpn1l-F1	*mPabpn1l*	siRNA	5’-CCACAGAUCUGUCUUUGUATT-3’
Pabpn1l-R1			5’-UACAAAGACAGAUCUGUGGTT-3’
Dis3l2-F1	*mDis3l2*	siRNA	5’-GCACCAAACUGAGCUACGATT-3’
Dis3l2-R1			5’-UCGUAGCUCAGUUUGGUGCTT-3’
m*Gapdh*-F	m*Gapdh*	Real-time PCR	5’-ACACTGAGGACCAGGTTGTCTC-3’
m*Gapdh*-R			5’-TACTCCTTGGAGGCCATGTAG-3’
miRNA cloning linker	Universal	3'-ligation RACE	5’-rAppCTGTAGGCACCATCAAT-NH_2-3’
RT+linker primer		3'-ligation RACE	5’-CTACGTAACGATTGATGGTGCCTACAG-3’
m*Gm4745*-3'-RACE-F	m*Gm4745*	3'-ligation RACE	5’-ATCTGCAGACAAGCTCTGGAAA-3’

m: mouse h: human

Supplementary Table S2. Antibody information.

**Protein name**	**Manufacture (catalog number)**	**Applications (working dilution)**
PABPN1	Bethyl (A303-523A)	IF (1:1000) WB (1:1000)
FITC-α-tubulin	Sigma (F2168)	IF (1:500) WB (1:2000)
HA	Cell Signaling (3724)	WB (1:2000)
FLAG	Sigma (F3165)	WB (1:2000)
LaminA/C	Invitrogen (MA3-1000)	WB (1:1000)
Phosphorylated RNA Pol II (Ser2P)	Abcam (ab5095)	IF (1:40000)
CDX2	BioGenex (AM392-5M)	IF (direct use)
NANOG	Cell Signaling (8822s)	IF (1:1000)
Rhodamine Phalloidin	Invitrogen (R415)	IF (1:1000)

Supplementary Table S3. Quality control of RNA-seq results (WT and si*Pabpn1* or

si*Dis3l2* embryos) (in a separate.xlsx file).

Supplementary Table S4. FPKMs of RNA-seq results (in a separate.xlsx file).

Supplementary Table S5. Spearman correlation coefficients among WT, si*Pabpn1*, and si*Dis3l2* embryos (in a separate.xlsx file).

Supplementary Table S6. Functional characterization of *Pabpn1*-regulated embryonic transcripts (in a separate.xlsx file).
